# MIPUP: minimum perfect unmixed phylogenies for multi-sampled tumors via branchings and ILP

**DOI:** 10.1093/bioinformatics/bty683

**Published:** 2018-08-08

**Authors:** Edin Husić, Xinyue Li, Ademir Hujdurović, Miika Mehine, Romeo Rizzi, Veli Mäkinen, Martin Milanič, Alexandru I Tomescu

**Affiliations:** 1 Department of Mathematics, London School of Economics and Political Science, London, UK; 2 Department of Computer Science, Helsinki Institute for Information Technology HIIT, University of Helsinki, Finland; 3 University of Primorska, UP IAM, Koper, Slovenia; 4 University of Primorska, UP FAMNIT, Koper, Slovenia; 5 Genome-Scale Biology Research Program, Research Programs Unit, Department of Medical and Clinical Genetics, Faculty of Medicine, University of Helsinki, Medicum, Helsinki, Finland; 6 Department of Computer Science, University of Verona, Verona, Italy

## Abstract

**Motivation:**

Discovering the evolution of a tumor may help identify driver mutations and provide a more comprehensive view on the history of the tumor. Recent studies have tackled this problem using multiple samples sequenced from a tumor, and due to clinical implications, this has attracted great interest. However, such samples usually mix several distinct tumor subclones, which confounds the discovery of the tumor phylogeny.

**Results:**

We study a natural problem formulation requiring to decompose the tumor samples into several subclones with the objective of forming a minimum perfect phylogeny. We propose an Integer Linear Programming formulation for it, and implement it into a method called MIPUP. We tested the ability of MIPUP and of four popular tools LICHeE, AncesTree, CITUP, Treeomics to reconstruct the tumor phylogeny. On simulated data, MIPUP shows up to a 34% improvement under the ancestor-descendant relations metric. On four real datasets, MIPUP’s reconstructions proved to be generally more faithful than those of LICHeE.

**Availability and implementation:**

MIPUP is available at https://github.com/zhero9/MIPUP as open source.

**Supplementary information:**

Supplementary data are available at Bioinformatics online.

## 1 Introduction

### 1.1 Background

Cancer is an evolutionary disease, with new mutations accumulating over time. Tumor genomes may carry up to thousands of mutations and one of the major challenges in cancer research is to distinguish between driver and passenger mutations. Furthermore, tumors are composed of several genetically distinct subpopulations, each harboring driver mutations. Identifying the set of mutations that belong to each subpopulation may help pinpoint which (gene) mutations are drivers. Moreover, understanding the order in which each driver mutation occurs will provide us with a more comprehensive view of tumor evolution. This can lead to a better understanding (Campbell *et al.*, 2008; Nik-Zainal *et al.*, 2012), and help in diagnosis and therapies (Newburger *et al.*, 2013).

High-throughput sequencing can offer a moderately-priced, genome-wide perspective of the mutations involved in the subclones of a tumor, as opposed to other more targeted methods such as single-cell sequencing, fluorescence *in situ* hybridization (FISH), or silver *in situ* hybridization (SISH) (Malikic *et al.*, 2015). However, the main drawback is that, by nature, more cell subpopulations are mixed in each sample.

Given such tumor high-throughput sequencing data, several questions pertain to it: what are the subpopulations of the tumor, in what proportion they occur, and what is the evolutionary relation among them. In case there is an evolutionary relation, the cell subpopulations are also called *subclones* of the tumor. Various computational methods have been proposed to address these questions, each answering a subset (or all) of them. Some methods assume as input a single sequencing sample from a tumor (Hajirasouliha *et al.*, 2014; Schwartz and Shackney, 2010; Strino *et al.*, 2013), whereas, as we will review in Section 1.2 below, other start the analysis with multiple samples.

In this paper we propose a multi-sample method for finding the tumor evolution, called MIPUP (minimum perfect unmixed phylogenies). MIPUP works by solving a problem equivalent to the Minimum-Split-Row problem proposed by Hajirasouliha and Raphael (2014), asking to minimally decompose the samples so that they form a perfect phylogeny. This phylogeny model is a common one, also used by e.g. Malikic *et al.* (2015), Popic *et al.* (2015), Jiao *et al.* (2014), El-Kebir *et al.* (2015). The method of this paper exploits a relation between perfect phylogenies and branchings in a directed acyclic graph from (Hujdurović *et al.*, 2018). Based on it, we give here a simple and efficient Integer Linear Programming (ILP) formulation for this problem.

We tested MIPUP against four other popular tools for discovering the tumor evolution, CITUP (Malikic *et al.*, 2015), LICHeE (Popic *et al.*, 2015), AncesTree (El-Kebir *et al.*, 2015), and Treeomics (Reiter *et al.*, 2017). We also tried testing against PASTRI (Satas and Raphael, 2017), but we could not run it (see the Supplementary Material). Under the perfect phylogeny assumption, over a range of scenarios (read coverage 100 1000 and 10000, a number of samples from 5 to 20) and 100 random trees simulated for each of these scenarios, MIPUP proved the most accurate in reconstructing the shape of the phylogenetic tree. This was measured as a proportion of how many of the original ancestor-descendant relations in the original tree were kept also in the reconstructed tree, as done also in (Popic *et al.*, 2015) and in (El-Kebir *et al.*, 2015). Our experiments show that, with respect to the overall two best performing tools among these four, MIPUP improves this metric by up to 34% for read coverage 100, by up to 11% for read coverage 1000, and by up to 20% for read coverage 10 000. In some cases, MIPUP reconstructs more than 92% of all relations, also on low coverage datasets. MIPUP also appeared resilient to a low number of loss of mutation events, which violate the perfect phylogeny assumption.

We also tested MIPUP and LICHeE on four real datasets. We manually inspected the output of both, and compared them to the reconstructions given in the papers the datasets were published in. We observe that, even though both tools output overall comparable trees, MIPUP’s results are generally more faithful to the original reconstructions, and require much less input parameters to fix.

### 1.2 Related work

In this section we review several methods that analyze multi-sample data from tumors. A few methods, such as Salari *et al.* (2013) and of van Rens *et al.* (2015), are primarily focused on improving the variant calling results in each sample. Many other methods are instead focused on reconstructing the evolutionary tree of the tumor using multiple samples. Among these latter methods, CITUP (Malikic *et al.*, 2015), LICHeE (Popic *et al.*, 2015) and AncesTree (El-Kebir *et al.*, 2015) assume only the variant allele frequencies (VAFs) of the mutations. Other methods, such as PhyloWGS (Deshwar *et al.*, 2015), Canopy (Jiang *et al.*, 2016), SPRUCE (El-Kebir *et al.*, 2016), also explicitly take into account copy-number aberrations.

Method CITUP works by exhaustively enumerating all possible trees with up to *N*_max_ nodes (where *N*_max_ is provided by the user), and decomposing each sample into several nodes of this tree. The fit between each sample and the tree is one minimizing a Bayesian information criterion on the VAF values. This fit is computed either exactly, with quadratic integer programming, or with a heuristic iterative method. The best tree is then output, together with the decompositions of each sample as nodes of this tree.

Method LICHeE also tries to fit the VAF values to a phylogenetic tree, but with an optimized search for such a tree. Mutations are first assigned to clusters based on their frequencies (a mutation can belong to more clusters). Then clusters are transformed to binary absence/presence vectors (with wildcards), based on two thresholds below which, and above which, the value is transformed into a 0 or a 1, respectively. Values in between are marked with a wildcard. The containment relation between these vectors induces a directed acyclic graph. Spanning trees of this graph are exhaustively enumerated, and the ones best compatible with the mutation frequencies are output.

Method AncesTree derives an ILP for the so-called VAF factorization problem (VAFF), namely the problem of determining the composition of each sample, including the number and proportion of clones in each sample, and a tree that describes the ancestral relationships between all clones. As the authors argue, this problem generalizes several previous formulations, including the above-mentioned (Hajirasouliha *et al.*, 2014; Jiao *et al.*, 2014; Malikic *et al.*, 2015; Strino *et al.*, 2013). The implementation behind AncesTree uses a more complex model than the VAFF problem, that also accounts for errors and is solved with a Mixed ILP.

El-Kebir et al. (2015) also argue that in the case of a single input sample, the VAFF problem generalizes the so-called Perfect Phylogeny Mixture Problem also proposed by Hajirasouliha and Raphael (2014), see (El-Kebir *et al.*, 2015, p. i64). Note that El-Kebir *et al.* (2015) propose an ILP for the initial VAFF problem, which is thus also applicable to the Perfect Phylogeny Mixture Problem. However, this problem is not equivalent to the problem underlying MIPUP, as it only asks for some decomposition of the samples into a perfect phylogeny, not necessarily a minimal one. Therefore, we cannot directly compare the efficiency of the ILP from this paper with the ILP of El-Kebir *et al.* (2015). See Table 1 for an overview of the advances relative to these two problems.

**Table 1.** Advances relative to the MCRS and the VAFF problems

	NP-hardness	Heuristic algorithms	ILPs
Hajirasouliha and Raphael (2014)	Only claimed	Only claimed	
Hujdurović *et al.* (2015, 2016)	Yes	Yes	
Hujdurović *et al.* (2018)	Strengthened to APX-hardness	Yes	Proved equivalence of problems MCRS and MUB (from Sec. 2.3)
This paper			Yes, based on MCRS equivalent to MUB
El-Kebir *et al.* (2015)	For VAFF problem, does not apply to MCRS		For VAFF problem, does not apply to MCRS

## 2 Materials and methods

### 2.1 Overview of the approach

**Fig. 1.**
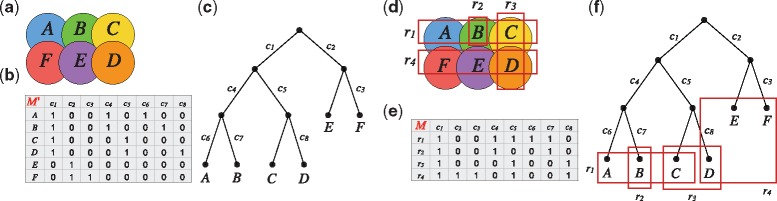
Overview of the approach. In (**a**) we illustrate a tumor with six subclones labelled A,…,F. In (**b**) we illustrate a binary matrix M′ such that every row is a tumor subclone, and every column is an SSNV found in at least one of the subclones (here the SSNVs are labeled $c_{1},\ldots ,c_{8}$). A 1 indicates presence and a 0 indicates absence of that SSNV in a subclone. In (**c**) we show the perfect phylogeny tree that gave rise to these patterns of mutations; here every subclone is a leaf of the tree and every SSNV labels an edge (and only one) of the tree. The SSNVs present in a subclone are the ones labeling the path from the root of the tree to the corresponding leaf. For example, the SSNVs present in subclone *A* are $\left\{c_{1},c_{4},c_{6}\right\}$, which are the same as the columns containing a “1” on row *A* in matrix *M* from (b). In practice, each sequencing sample may generally contain more than a single subclone of a tumor. In (**d**) we show four samples $r_{1},\ldots ,r_{4}$ sequenced from the tumor, some combining more than one subclone. In (**e**) we show the binary matrix *M* indicating presence/absence of the SSNVs in each of these four samples. Observe that each row *r_i_* of *M* is the bitwise OR of the binary rows of M′ corresponding to the subclones that are in sample *r_i_*. For example, sample *r*_1_ contains subclones *A*, *B*, *C*, and thus row *r*_1_ of *M* is the bitwise OR of rows *A*, *B*, *C* of M′. Figure 1**f** shows the same perfect phylogeny tree as in (c), in which we again mark the phylogeny nodes being combined in each sample *r_i_*. Matrix *M* is the input to our problem, and matrix M′ and the phylogeny tree corresponding to M′ are the unknowns that must be reported in output

In this section we give an informal overview of our approach. We refer the reader to Figure 1 for a visual overview.

Assume we obtained samples $r_{1},\ldots ,r_{m}$ from a tumor. Using a somatic point mutation caller, such as VarScan 2 (Koboldt *et al.*, 2012), we can detect the somatic single nucleotide variants (SSNVs) present in each sample and derive their VAF values from the read alignments over their positions. Denote these SSNVs by $c_{1},\ldots ,c_{n}$. We then build a binary matrix *M* with rows labeled $r_{1},\ldots ,r_{m}$ and columns labeled $c_{1},\ldots ,c_{n}$, such that $M_{i,j}=1$ if and only if the VAF value of SSNV *c_j_* in sample *r_i_* is greater or equal to a given threshold *t*.

Matrix *M* is the input to our problem. From it, we would like to infer (i) the individual subclones of the tumor making up each sample *r_i_* (i.e., the binary pattern of SSNVs in each such subclone) and (ii) the evolutionary relation among these unknown subclones.

**Fig. 2.**
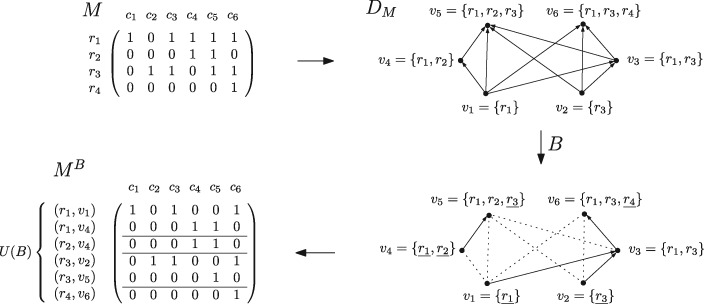
An example of a binary matrix *M*, its containment digraph *D_M_*, a branching *B*, and the resulting *B*-split *M^B^* of *M*. The row split *M^B^* is an optimal solution to the MCRS problem given *M*. Pairs (*r*, *v*) for which *r* is underlined as an element of *v* in the figure showing *B* are exactly the uncovered elements with respect to *B*. Figure adapted from (Hujdurović *et al.*, 2018)

Let us now make these notions more precise. In this paper we consider the model and problem formulation proposed by Hajirasouliha and Raphael (2014). This considers as evolutionary relation among the tumor subclones the so-called *perfect phylogeny* model, in line with previous studies such as (El-Kebir *et al.*, 2015; Jiao *et al.*, 2014; Malikic *et al.*, 2015; Popic *et al.*, 2015). This assumes that (i) all mutations in the parent cells are passed to the descendants, and (ii) once a mutation occurs at a particular site, it does not occur again at that site (the “infinite sites assumption”). Being mixtures of subclones of the tumor, the rows of *M* may not necessarily form a perfect phylogeny. Thus, we would like to split each row *r_i_* of *M* into a set of rows *R_i_* so that the resulting matrix M′ does correspond to a perfect phylogeny. (See Definition 2.2 for a formal definition of the *split* operation, and Figure 2 for an example of a matrix *M* and a matrix *M^B^* obtained by splitting the rows of *M*.) Hajirasouliha and Raphael (2014) proposed to perform this split so that the resulting matrix is “minimal”. Such parsimony criterion is often employed when modeling real-life problems, and it is one of the most basic investigations one can perform.

More specifically, Hajirasouliha and Raphael (2014) proposed that M′ has the minimum number of rows. In terms of perfect phylogeny trees, this means that we are looking to split each sample into a collection of subclones forming a perfect phylogeny, and the total number of subclones from all samples is minimum. We will call this problem MinimumConflict-FreeRowSplit (MCRS), see Section 2.2.

Hajirasouliha and Raphael (2014) claimed that the MCRS problem is NP-hard (and gave an incorrect proof), and in (Hujdurović *et al.*, 2015, 2016) a correct hardness proof was given. Hujdurović *et al.* (2016) also proposed a polynomial-time heuristic algorithm for it based on coloring co-comparability graphs and tested it on real samples.

As opposed to the above heuristic algorithm, in this paper we propose an exact algorithm for the MCRS problem. We obtain this by using a recent result from (Hujdurović *et al.*, 2018) showing that the problem is equivalent to a problem related to finding an optimal branching in a directed acyclic graph. A *branching* is a subgraph in which every vertex has out-degree at most 1. We formally describe this correspondence in Section 2.3. Using this branching formulation, we then show in Section 2.4 that the MCRS problem can be expressed using ILP, and solve it using the CPLEX ILP solver.

See Table 1 for a summary of these results.

### 2.2 Problem formulation

A *binary matrix*$M\in {\left\{0,1\right\}}^{m\times n}$ is a matrix having *m* rows and *n* columns, and all entries 0 or 1. Each row of such a matrix is a vector in ${\left\{0,1\right\}}^{n}$; each column is a vector in ${\left\{0,1\right\}}^{m}$. We will denote by $R_{M}={\left(r_{i}\right)}_{1\leq i\leq m}$ and $C_{M}={\left(c_{j}\right)}_{1\leq j\leq n}$ the families of rows and columns of *M*, respectively. The entry of *M* at row *r_i_* and column *c_j_* will be denoted by $M_{i,j}$ or $M_{r_{i},j}$ when appropriate. For brevity, we will often write “the number of distinct rows (resp., columns) of *M*” to mean “the maximum number of pairwise distinct rows (resp., columns) of *M*”. Two rows (resp., columns) are considered distinct if they differ as binary vectors. All binary matrices in this paper will be assumed to contain no row in which all entries are 0.

Definition 2.1. Given a matrix *M*, three distinct rows *r*, r′, r″ of M and two distinct columns *i* and *j* of *M*, we denote by M[(r,r′,r″),(i,j)] the 3 × 2 submatrix of *M* formed by rows *r*, r′, r″ and columns *i*, *j* (in this order). Two columns *i* and *j* of a binary matrix *M* are said to be in conflict if there exist rows r,r′,r″ of *M* such that

$$M[(r,r\prime ,r\prime \prime ),(i,j)]=\left(\begin{array}{cc} 1 & 1\\ 1 & 0\\ 0 & 1 \end{array}\right).$$

We say a binary matrix *M* is conflict-free if there exist no two columns of *M* that are in conflict.

The rows of a binary matrix *M* are the leaves of a perfect phylogenetic tree if and only if *M* is conflict-free, see (Estabrook *et al.*, 1975; Gusfield, 1997). Moreover, if this is the case, then the corresponding phylogenetic tree can be retrieved from *M* in time linear in the size of *M* (Gusfield, 1991). As such, we formulate our problems just in terms of finding optimal conflict-free matrices.

**Fig. 3.**
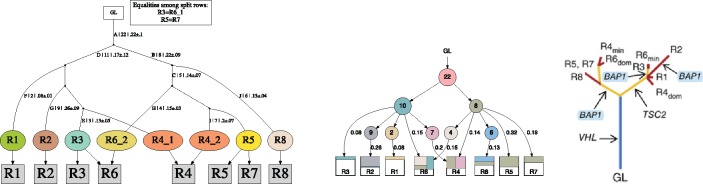
From left to right: the output of MIPUP, LICHeE and the tree reported in the original publication, for dataset RMH008 from (Gerlinger *et al.*, 2014). The last row of square gray nodes in the trees of MIPUP are the original samples. The oval nodes are the rows in which the input matrix is split. Notice that, due to our tree building algorithm, they are drawn as leaves of the phylogeny. However, if their in-coming edge has no label (i.e., no mutations occurring on that edge) then they are actually internal nodes of the evolution, recall Remark 2.1. For example, node R1 is internal to the evolution. Arrows indicate the composition of the original samples in terms of split rows. The legend contains the equalities among split rows; only one split row in each equality class is a node of the tree

Remark 2.1. We are following here the formalism on perfect phylogenies from (Gusfield, 1991). Namely, each row of a matrix is a leaf of the phylogenetic tree, and columns label edges. However, a leaf whose in-coming edge has no label is in fact an internal node of the evolution, that is, it has no “private” mutations. See for example Figure 1c where leaves C and E have no labels on the in-coming edges. We follow the same formalism in the trees output by MIPUP, see Figure 3.

Definition 2.2. Let $M\in {\left\{0,1\right\}}^{m\times n}$. Label the rows of *M* as $r_{1},r_{2},\ldots ,r_{m}$. A binary matrix $M\prime \in {\left\{0,1\right\}}^{m^{\prime }\times n}$ is a row split of *M* if there exist a partition of the set of rows of M′ into m sets $R_{1},R_{2},\ldots R_{m}$ such that for all i∈{1,…,m}, *r*_i_ is the bitwise OR of the binary vectors in *R*_i_. The set *R*_i_ of rows of M′ is said to be the set of split rows of row *r*_i_ (with respect to M′).

For simplicity, we defined a row split as a binary matrix M′ for which a suitable partition of rows exists. However, throughout the paper we will make a slight technical abuse of this terminology by considering any row split M′ of *M* as already equipped with an arbitrary (but fixed) partition of its rows $R_{1},\ldots ,R_{m}$ satisfying the above condition.

We denote by γ(M) the minimum number of rows in a conflict-free row split M′ of *M*. Formally, the minimum conflict-free row split problem is defined as follows:

**Table:** 

MinimumConflict-FreeRowSplit (MCRS):	
*Input:*	A binary matrix *M*.
*Task:*	Compute γ(M) and find a conflict-free row split M′ of *M* with γ(M) rows.

### 2.3 The branching formulation

In this section we review the formulation from (Hujdurović *et al.*, 2018) of the MCRS problem in terms of branchings in a directed acyclic graph (DAG). We refer the reader to (Hujdurović *et al.*, 2018) for the proof of this equivalence. In Section 2.4 we will use this formulation to write an ILP for the problem.

Definition 2.3. Let D=(V,A) be a DAG. A branching of *D* is a subset *B* of *A* such that (*V*, *B*) is a directed graph in which for each vertex *v* there is at most one arc leaving *v*.

The following construction can be performed on any given binary matrix *M* and results in a DAG. Given a column $c_{j}\in C_{M}$, the *support of c_j_* is the set defined as $\left\{r_{i}\in R_{M}:M_{i,j}=1\right\}$ and denoted by ${\text{supp}}_{M}(c_{j})$. Given a binary matrix $M\in {\left\{0,1\right\}}^{m\times n}$, the *containment digraph D_M_* of *M* is the DAG with vertex set $V=\{{\text{supp}}_{M}(c):c\in C_{M}\}$ and arc set A={(v,v′):v,v′∈V∧v⊂v′} where ⊂ is the relation of proper inclusion of sets.

Let $M\in {\left\{0,1\right\}}^{m\times n}$ be a binary matrix, let $D_{M}=(V,A)$ be the containment digraph of *M*, and let *B* be a branching of *D_M_*. For a vertex v∈V, we denote by $N_{B}^{-}(v)$ the set of all vertices v′∈V such that (v′,v)∈B. *A source* of *B* is a vertex not entered by any arc of *B*. For a vertex v∈V, an element r∈v (that is, a row of *M*) is said to be *covered* in *v* with respect to *B* (or just *B-covered*) if $r\in \cup N_{B}^{-}(v)$. Analogously, we say that r∈v is *uncovered* in *v* with respect to *B* if *r* is not covered in *v*. A *B-uncovered pair* is a pair (*r*, *v*) such that *r* is a row of *M*, *v* is a vertex of *D_M_* (that is, the support of a column of *M*), r∈v, and *r* is uncovered in *v* with respect to *B*. For a row *r* of *M*, we will denote by $U_{B}(r)$ the set of all *B*-uncovered pairs with first coordinate *r*, and by *U*(*B*) the set of all *B*-uncovered pairs. We illustrate these notions in Figure 2, where two branchings *B*_1_ and *B*_2_ of the arc set of *D_M_* are depicted, together with uncovered pairs (*r*, *v*) with respect to each of the two branchings.

We denote with β(M) the minimum number of elements in *U*(*B*) over all branchings *B* of *D_M_*. The corresponding optimization problem is the following:

**Table:** 

MinimumUncoveringBranching (MUB):	
*Input:*	A binary matrix *M*.
*Task:*	Compute β(M) and find a branching *B* of *D_M_* with |U(B)|=β(M).

The announced equivalence between the MCRS and the MUB problems is captured in the following result.

Theorem 2.1: Hujdurović *et al.* (2018). For every binary matrix $M\in {\left\{0,1\right\}}^{m\times n}$ with exactly *k* distinct columns, we have γ(M)=β(M). Moreover, for any branching *B* of *D_M_* can be transformed in time O(mkn) to a conflict-free row split of M with exactly |U(B)| rows.

The following notion of *B-split* specifies how each branching *B* corresponds to a row split of *M*.

Definition 2.4. Let *M* be a binary matrix with rows $r_{1},\ldots ,r_{m}$ and columns $c_{1},\ldots ,c_{n}$. For a branching *B* of *D_M_*, we define the *B*-split of *M*, denoted by *M^B^*, as the matrix with rows indexed by the elements of the set *U(B)*, and columns $c\prime_{1},\ldots ,c\prime_{n}$, as follows. Let $V=V(D_{M})$ and for all j∈{1,…,n}, let $v_{j}={\text{supp}}_{M}(c_{j})$ (so $v_{j}\in V$). For a vertex v∈V, we denote by $B^{+}(v)$ the set of all vertices in *V* reachable by a directed path from *v* in (*V*, *B*) [note that $v\in B^{+}(v)]$. For all (r,v)∈U(B) and all j∈{1,…,n}, set:

$$M_{(r,v),j}^{B}=\{\begin{array}{cc} 1, & \;if\;v_{j}\in B^{+}(v);\\ 0, & \;otherwise. \end{array}$$

See Figure 2 for an example of a binary matrix *M* with two branchings *B*_1_ and *B*_2_ of its containment digraph and the corresponding row splits.

The proof of Theorem 2.1 from (Hujdurović *et al.*, 2018) shows that the *B*-split of *M* is conflict-free and has |U(B)| rows. This means that if we have a branching minimizing |U(B)|, then the *B*-split of this branching is an optimal solution for the MCRS problem.

### 2.4 ILP formulation

The notion of *B*-split can be used to transform an optimal solution to the problem of computing one of the parameters {β,γ} to an optimal solution for the other parameter. The problem formulation in terms of *β* is directly expressible in terms of packing and covering constraints, and thus leads to a natural integer programming formulation of the MUB problem. We will express the ILP only in terms of finding the value β(M). However, the optimal branching attaining this value can be trivially retrieved from the values of the variables in an optimal solution of the ILP.

Remark 2.2. It is easy to check that the decision version of the MCRS problem is in NP and thus admits a polynomially-sized certificate. Furthermore, since Integer Linear Programming is NP-hard, it follows that there exists a polynomially sized ILP formulation of the MCRS problem. However, applying Theorem 2.1 allows to obtain a direct and simple polynomially-sized ILP formulation for it, which will also turn out to be efficient in practice.

Let *M* be the input binary matrix to the problem, and let $D_{M}=(V,A)$ be its containment digraph. Our goal is to find a branching *B* of *D_M_* minimizing the number of elements in *U*(*B*). We introduce the following binary variables:

- for every edge (u,v)∈A, we introduce a variable $x_{u,v}$ with the intended meaning that $x_{u,v}=1$ if and only if (u,v)∈B;
- for all v∈V and for all r∈v, we introduce a variable $y_{r,v}$, meaning $y_{r,v}=1$ if and only if *r* is uncovered in *v* with respect to *B*.

Consider the following integer program: $\min\sum_{v\in V}\sum_{r\in v}y_{r,v}$ subject to

(1) $$\sum_{(u,v)\in A}x_{u,v}\leq 1\;\;\;\forall u\in V$$

(2) $$y_{r,v}+\sum_{u\in N_{A}^{-}(v)\;:\;r\in u}x_{u,v}\geq 1\;\forall r\in v\in Vx_{u,v},y_{r,v}\;\text{binary}$$

Theorem 2.2. The optimal value of the above integer program is β(M).

PROOF. Let OPT denote the optimal value of the above ILP.

First, we prove that OPT≤β(M). Let *B* be a branching of *D_M_* such that |U(B)|=β(M). Define a binary vector $x\in {\left\{0,1\right\}}^{A}$ by setting

$$x_{u,v}=\{\begin{array}{cc} 1, & \;\text{if}\;(u,v)\in B;\\ 0, & \;\text{otherwise}. \end{array}$$

For every v∈V and every r∈v set $y_{r,v}=1$ if and only if *r* is uncovered in *v* with respect to *B*. The objective function value at (*x*, *y*) equals to the sum, over all *v*, of the number of uncovered elements in *v* with respect to *B*, that is, the size of *U*(*B*). The definition of a branching implies that constraints (1) are satisfied. Consider now a constraint of type (1). Let v∈V and r∈v. If $y_{r,y}=1$, then the constraint holds due to the non-negativity of the *x*-variables. If $y_{r,v}=0$, then *r* is covered in *v* with respect to *B*. This implies that there exists an arc (u,v)∈B such that r∈u. Since (u,v)∈B, it holds $x_{u,v}=1$ and thus the constraint is satisfied in this case. It follows that (*x*, *y*) is a feasible solution of the ILP with objective function value |U(B)|, therefore OPT≤|U(B)|=β(M).

The proof of the other inequality is similar. Let (*x*, *y*) be an optimal solution to the ILP and let *B* be the set of arcs (u,v)∈A such that $x_{u,v}=1$. Constraints (1) guarantee that *B* is a branching of *D_M_*. Constraints (2) and the optimality of (*x*, *y*) imply that for all v∈V and all r∈v, we have $y_{r,v}=1$ if and only if $\sum_{u\in N_{A}^{-}(v)\;:\;r\in u}x_{u,v}=0$. Indeed, if the above sum is at least 1, then setting $y_{r,v}$ to 0 would result in a feasible solution with strictly smaller objective function value. Therefore, $y_{r,v}=1$ if and only if (u,v)∈B for all $u\in N_{A}^{-}(v)$ such that r∈u, which is in turn equivalent to the condition r∈∪v′∈NB−(v)v′, that is, *r* is uncovered in *v* (with respect to *B*). It follows that the objective function value at (*x*, *y*) equals the total number of uncovered pairs, that is, the size of *U*(*B*). We conclude that *B* is a branching such that |U(B)|=OPT, which implies β(M)≤OPT. □

The above integer program has $p=|A|+\sum_{v\in V}|v|$ binary variables and $q=|V|+\sum_{v\in V}|v|$ constraints. In terms of the binary matrix *M*, the numbers of variables and constraints can be described as: p=ℓ+o and q=k+o, where *k*, ℓ, and *o* denote the number of columns, the number of comparable pairs of columns (with respect to the containment relation), and the number of ones in the matrix obtained by taking from *M* exactly one copy from each set of identical columns, respectively. If *M* is *m *×* n*, then the number of variables is O(n(m+n)) and the number of constraints is *O*(*mn*).

### 2.5 Implementation

MIPUP is implemented in Java and uses the CPLEX ILP solver. MIPUP can report all optimal solutions, or at most a user-provided number of optimal solutions.

The input format is the same as for LICHeE, namely a matrix with VAF values of each SSNV in each sample. As input we also assume a threshold *t* to transform VAF values into binary ones. LICHeE applies a further filtering to the input matrix, namely removing those *weak* SSNVs whose binary presence/absence pattern in the samples appears strictly less than *k* times (option minClusterSize) in the entire matrix (default *k *=* *2). We also provide a Python script that, given *t* and *k*, filters the matrix in this manner.

Apart from an optimal conflict-free row split binary matrix, MIPUP also outputs the perfect phylogeny tree corresponding to it. We label each edge of the tree with the set of mutations that occurred along the edge. The label format is S|n|mean±std, where *S* is an internal name for the group of mutations (the mutations corresponding to each group are output in a separate file), *n* is the cardinality of *S*, *mean* is the mean value of their VAF values, in all samples, and *std* is the standard deviation of their VAF values. See the caption of Figure 3 for further details on the layout of the phylogenetic trees.

## 3 Experiments

### 3.1 Simulated data

We performed an evaluation of simulated data as done in (El-Kebir *et al.*, 2015) and in (Popic *et al.*, 2015). Our evaluation pipeline is freely available at https://github.com/huanyannizu/Data-simulation-and-evaluation-in-MIPUP. We created uniformly at random a tree with *c* nodes (i.e., clones), and randomly chosen a node as root. This was done using an algorithm based on Prüfer’s encoding of a labeled tree (Prüfer, 1918). We randomly assigned *n* mutations to the nodes of this tree, making sure each node gets at least one mutation. Our main experiments are with *c *=* *10 and *n *=* *100, as in (El-Kebir *et al.*, 2015). In order to see how the tools scale, we also tested MIPUP, LICHeE, and Treeomics with *c *=* *20, *n *=* *200 and *c *=* *30, *n *=* *300.

Note that, under the perfect phylogeny assumption, the mutations in a node must be iteratively propagated to all descendants of a node. To test also loss of mutation events, we added a further parameter d∈{0,1,…,9} that denotes the number of times one of these propagation events of a mutation in some node *v* (that may have originated in *v* or in an ancestor of *v*) is *not* propagated to a child *u* of *v* (and thus to none of the descendants of *u*). Note that *d *=* *0 corresponds to the perfect phylogeny assumption. We then assigned to each node a random cell population size between 100 and 200.

We created a number of *m* samples from the tree as follows. Each sample randomly selects 2–4 nodes of the tree, and will include all cells and mutations in those nodes. As in (El-Kebir *et al.*, 2015), we then created three matrices, *U*, *B*, *F*: *usage* matrix $U\in R^{m\times c}$ is such that an entry (*r_i_*, *c_j_*) contains the fraction of cells of clone *c_j_* out all the cells in sample *r_i_*; *clonal* matrix $B\in {\left\{0,1\right\}}^{c\times c}$ is such that an entry (*c_i_*, *c_j_*) equals 1 iff *c_i_* = *c_j_*, or *c_i_* is a descendant of *c_j_* in the tree; *VAF value* matrix $F\in R^{m\times c}$ equals $\frac{1}{2}UB$ and contains the true VAF values of all mutations in each clone. See (El-Kebir *et al.*, 2015, Fig. 1) for details. We then unpack matrix *F* into $F_{unpack}\in R^{m\times n}$, which has a column for each mutation, so that the column corresponding to mutation *m_j_* from clone *c_k_* is the same as column *c_j_* of *F*.

Note that tools MIPUP, LICHeE and CITUP accept in input VAF values. However, tools AncesTree and Treeomics require reads counts. For this reason, we simulated reads counts as in done in (El-Kebir *et al.*, 2015). Given a read coverage a∈{100,1000,10 000}, we draw the number of reads containing mutation *m_j_* in sample *r_i_* as $y_{r_{i},m_{j}}∼Poiss(a)$. We then draw the number of reads containing the variant allele as $x_{r_{i},m_{j}}∼Bionomial(y_{r_{i},m_{j}},F_{r_{i},m_{j}})$. The number of reads containing the reference allele is $y_{r_{i},m_{j}}-x_{r_{i},m_{j}}$. The values $x_{r_{i},m_{j}}/y_{r_{i},m_{j}}$ are thus noisy VAF values that are used as input also for MIPUP, LICHeE, CITUP.

For each *m* and each read coverage a∈{100,1000,10 000}, we simulated 100 trees and ran the tools on the above noisy read counts and VAF values. For the main scenario (c,n)=(10,100) [as in (El-Kebir *et al.*, 2015)], we chose m∈{5,10,15,20}. For (c,n)∈{(20,200),(30,300)}, where we were interested mainly in the running times, we ran MIPUP, LICHeE, and Treeomics only for *m *=* *5 samples.

We evaluated how well the tools are able to reconstruct the original tree, as done in (Popic *et al.*, 2015) and (El-Kebir *et al.*, 2015). Given the original tree, and given two mutations *m_i_* and *m_j_* in clones *c_i_* and *c_j_*, we say that *m_i_* is an *ancestor* (resp. *descendant*) of *m_j_* if *c_i_* is an *ancestor* (resp. *descendant*) of *c_j_*. An *AD pair* is an ordered pair (*m_i_*, *m_j_*) of mutations such that *m_i_* is an ancestor of *m_j_*. Note that two mutations in the same node are not an AD pair. Given an output tree reported by each tool, we computed the fraction of AD pairs in the original tree that were present in the output tree.

Note that MIPUP, CITUP, and Treeomics can report more output “best” trees. (In MIPUP’s case, unless otherwise stated, we output all optimal trees.) In this case, we report three results for them, “Best”—the tree achieving the best results under our metric; “Avg”—the average metric over all reported trees, and “Std”—their standard deviation. Note that results “Best” are usually unattainable in practice.

The results for (c,n)=(10,100) are in Table 2. For none, or very few, losses of mutation (d≤2) MIPUP is generally the best performing tool. As *d* increases, Treeomics becomes the best performing tool. However, for large values of *d*, the results of all tools are significantly worse than under the perfect phylogeny assumption (*d *=* *0). See Table 2 for results for *d *=* *9 and the Supplementary Material for all other values of *d*. While it appear that Treeomics produces better results as we increase the number of loss mutation events, it is worth noting that all models (MIPUP, CITUP, Treeomics and LICHeE) do assume the perfect phylogeny model.

**Table 2.** The fraction of original AD pairs kept in the output trees by each method, for (c,n)=(10,100) and a number of d∈{0,1,2,9} of loss of mutation events

*d *=* *0		MIPUP			LICHeE	Treeomics			CITUP			Ances Tree
*m*	cov.	Best	Avg	Std		Best	Avg	Std	Best	Avg	Std	
d=0												
5	100	0.734	**0.718**	0.04	0.672	0.702	0.681	0.02				0.111
	1000	0.691	0.665	0.06	**0.669**	0.642	0.611	0.03	0.402	0.390	0.08	0.076
	10000	0.720	**0.702**	0.04	0.680	0.654	0.614	0.04	0.383	0.368	0.09	0.084
10	100	0.871	**0.855**	0.04	0.734	0.825	0.810	0.01				0.017
	1000	0.896	**0.881**	0.06	0.878	0.829	0.789	0.04	0.431	0.431	0.00	0.016
	10000	0.878	**0.856**	0.06	0.843	0.758	0.710	0.05	0.397	0.392	0.14	0.018
15	100	0.897	**0.888**	0.03	0.732							
	1000	0.908	**0.902**	0.04	0.893							
	10000	0.924	**0.918**	0.04	0.909							
20	100	0.934	**0.918**	0.05	0.684							
	1000	0.932	**0.929**	0.04	0.909							
	10000	0.949	**0.945**	0.04	0.928							
d=1												
5	100	0.650	**0.621**	0.07	0.541	0.637	0.619	0.02				0.095
	1000	0.699	**0.680**	0.04	0.647	0.671	0.631	0.04	0.433	0.413	0.14	0.078
	10000	0.663	**0.644**	0.04	0.594	0.619	0.593	0.03	0.412	0.396	0.11	0.089
10	100	0.773	**0.757**	0.03	0.633	0.756	0.737	0.02				0.016
	1000	0.738	**0.720**	0.05	0.689	0.718	0.674	0.04	0.435	0.433	0.12	0.015
	10000	0.792	**0.775**	0.05	0.715	0.730	0.650	0.07	0.459	0.458	0.20	0.015
15	100	0.799	**0.785**	0.03	0.630							
	1000	0.812	**0.801**	0.04	0.764							
	10000	0.832	**0.827**	0.02	0.787							
20	100	0.826	**0.819**	0.02	0.645							
	1000	0.845	**0.842**	0.03	0.797							
	10000	0.828	**0.825**	0.03	0.774							
d=2												
5	100	0.555	**0.537**	0.03	0.443	0.556	0.525	0.03				0.095
	1000	0.603	**0.577**	0.05	0.507	0.581	0.551	0.02	0.368	0.343	0.11	0.050
	10000	0.619	**0.585**	0.06	0.520	0.618	0.573	0.04	0.412	0.390	0.14	0.047
10	100	0.691	0.671	0.04	0.577	0.720	**0.687**	0.03				0.017
	1000	0.651	**0.633**	0.04	0.576	0.663	0.610	0.05	0.400	0.399	0.10	0.014
	10000	0.684	**0.665**	0.05	0.594	0.661	0.589	0.07	0.434	0.426	0.13	0.014
15	100	0.692	**0.679**	0.04	0.555							
	1000	0.700	**0.693**	0.03	0.651							
	10000	0.735	**0.722**	0.06	0.677							
20	100	0.670	**0.660**	0.03	0.534							
	1000	0.733	**0.729**	0.02	0.686							
	10000	0.683	**0.681**	0.01	0.645							
d=9												
5	100	0.223	0.197	0.20	0.158	0.307	**0.277**	0.03				0.019
	1000	0.196	0.170	0.17	0.133	0.310	**0.274**	0.03	0.101	0.089	0.04	0.008
	10000	0.228	0.199	0.20	0.164	0.344	**0.323**	0.02	0.127	0.112	0.05	0.012
10	100	0.178	0.165	0.16	0.139	0.336	**0.308**	0.03				0.005
	1000	0.201	0.182	0.18	0.167	0.416	**0.376**	0.04	0.073	0.071	0.00	0.003
	10000	0.255	0.237	0.24	0.216	0.530	**0.461**	0.06	0.099	0.099	0.00	0.004
15	100	0.187	**0.182**	0.18	0.160							
	1000	0.219	**0.210**	0.21	0.192							
	10000	0.195	**0.190**	0.19	0.173							
20	100	0.204	**0.201**	0.20	0.174							
	1000	0.186	**0.183**	0.18	0.173							
	10000	0.215	**0.213**	0.21	0.198							

*Notes:* Empty cells correspond to scenarios where the tools could not run (see the Supplementary Material for details). The best average results are in bold.

Manually checking the outputs, we observe that one reason why MIPUP performs better is that other tools (especially CITUP and AncesTree) combine more parent-child nodes of the initial tree into a single node, and thus are not able to recover the initial AD pairs from these nodes (for example, in a few cases, AncesTree outputs a tree made up of a single node).

As seen from Table 3, MIPUP (even when outputting all optimal solutions) and LICHeE generally run in less than two seconds, and Treeomics generally runs in less than one minute. The running time of CITUP and AncesTree is an order of magnitude higher and more variable.

**Table 3.** Top: The running time (in seconds) of MIPUP, MIPUP limited to outputting only one optimal solution (MIPUP - one), LICHeE, Treeomics, CITUP and AncesTree, for (c,n)=(10,100). Bottom: The running time of MIPUP, MIPUP – one, LICHeE, Treeomics for (c,n)∈{(20,200),(30,300)} and *m *=* *5 samples

		MIPUP		MIPUP - one		LICHeE		Treeomics		CITUP		AncesTree	
*m*	coverage	Avg	Std	Avg	Std	Avg	Std	Avg	Std	Avg	Std	Avg	Std
5	100	0.22	0.06	0.17	0.02	1.30	0.09	5.05	0.53			9.85	59.48
	1000	0.21	0.03	0.17	0.02	1.32	0.09	5.05	0.48	111.74	51.20	14.16	44.71
	10000	0.21	0.03	0.17	0.02	1.31	0.09	5.33	0.60	118.15	63.62	12.93	10.56
10	100	0.23	0.05	0.17	0.02	1.36	0.09	37.77	1.97			125.58	221.44
	1000	0.23	0.05	0.18	0.05	1.39	0.12	49.77	18.81	601.58	307.60	182.54	282.59
	10000	0.22	0.03	0.17	0.02	1.36	0.09	56.88	20.02	693.58	377.32	143.07	258.04
15	100	0.29	0.20	0.16	0.02	1.36	0.09						
	1000	0.24	0.09	0.17	0.02	1.39	0.09						
	10000	0.23	0.02	0.17	0.02	1.38	0.09						
20	100	0.27	0.11	0.18	0.02	1.39	0.09						
	1000	0.24	0.03	0.17	0.02	1.42	0.11						
	10000	0.24	0.04	0.17	0.03	1.40	0.11						


	MIPUP		MIPUP - one		LICHeE		Treeomics	
	Avg	Std	Avg	Std	Avg	Std	Avg	Std
20 nodes, 200 mutations	0.29	0.17	0.18	0.02	1.40	0.11	6.32	0.86
30 nodes, 300 mutations	0.36	0.21	0.18	0.02	1.46	0.14	7.21	0.95

### 3.2 Real data

We experimented on four real datasets: ultra-deep-sequencing of clear cell renal cell carcinoma (ccRCC) (Gerlinger *et al.*, 2014) (also analysed by LICHeE), high-grade serous ovarian cancer (HGSC) by (Bashashati *et al.*, 2013), breast cancer xenoengraftment in immunodeficient mice (Eirew *et al.*, 2015) and (four) clonally related uterine leiomyomas (Mehine *et al.*, 2015). The first three datasets are public and were also considered by Popic *et al.* (2015). The public datasets can also be found in the MIPUP repository, together with the experiment results, and the scripts and parameters used to run them. We ran only MIPUP and LICHeE on these real datasets.

In Supplementary Table S1 we show an overview of the sizes of the input matrices. In Figure 3 we show the results on the RMH008 samples from the ccRCC study of Gerlinger *et al.* (2014). The results on other samples are shown and discussed in the Supplementary Material.

Even though the results of LICHeE and MIPUP generally agree, in many instances there are many slight differences among them, and MIPUP is generally closer to the original phylogenies proposed in the papers analyzing the datasets. For example, on sample RMH008 from Figure 3, MIPUP reports that samples R6 and R4 are combinations of two phylogeny nodes, which lie on a tree branch together with R1, R2, and R3, and on a tree branch together with R5, R7, and R8. This is in line with LICHeE’s prediction and with (Gerlinger *et al.*, 2014). However, there are some differences: in line with (Gerlinger *et al.*, 2014) (right branch), MIPUP reports that R6 is made up of some SSNVs common only to R3, as opposed to all of R1, R2, R3 in LICHeE’s case. It also reports that R6 is made up of SSNVs common to R4, R5, R7 (node R6_2), in line with (Gerlinger *et al.*, 2014) (left branch), as opposed to all of R4, R5, R7, R8 in LICHeE’s case.

Moreover, in order to run LICHeE accurately, the user must guess many input parameters, while in MIPUP’s case the user must fix only one, the threshold for converting a VAF value into a binary one. In fact, for many of the samples in the ccRCC dataset analyzed by LICHeE, the input parameters were chosen by LICHeE’s authors as different from the default values.

## 4 Conclusion

MIPUP solves exactly and efficiently a natural problem related to minimally unmixing sequencing samples so that they fit a perfect phylogeny. We tested MIPUP against a large number of competing tools, and shown that MIPUP reconstructs the original tree (under the ancestor-descendant metric) significantly better. On real data, MIPUP generally has more faithful reconstructions than LICHeE, with much less input parameters to guess correctly. On the methodological side, MIPUP’s novelty is in the reduction of a phylogeny problem to a branching problem and in the search for the optimum phylogeny embedded in the ILP formulation itself.

We believe that MIPUP’s performance stems from two ingredients. First, from a much simpler problem formulation. Second, MIPUP’s most significant increase in performance is for low read coverage, where noisy data can have greater effects on methods using VAF values explicitly. MIPUP transforms VAF values to binary ones. Since MIPUP does not try to reconstruct the proportion of each clone in each sample, but only their ancestral relation, this suggests that transforming VAF values into binary ones is actually a more resilient choice for this scenario and thus an advantage for MIPUP.

## Supplementary Material

Supplementary Material
