# Supplementary material for: MIPUP: minimum perfect unmixed phylogenies for multi-sampled tumors via branchings and ILP
Source: Bioinformatics. 2018 Aug 8;35(5):769–77. doi: 10.1093/bioinformatics/bty683 (PMC6394401; doi:10.1093/bioinformatics/bty683)
Supplement: Supplementary Material [file bty683_supplementary_material.pdf]

MIPUP: Supplementary material

1 Further details on simulated data

1.1 Running the tools

In this section we give more details on how we ran each tool.

MIPUP MIPUP converts the VAF values into binary ones using a threshold `t`. We set `t` to 0.05.

LICHeE For LICHeE we set the two parameters `-maxVAFabsent` and `-minVAFpresent` to 0.05 and used default values for all other parameters.

AncesTree We used all default parameters. AncesTree outputs a `.txt` file and a `.dot` file with the tree structure. Sometimes AncesTree reports in the `.txt` file more solutions, but it still generates a single `.dot` and thus a single output tree. We used only this tree in our evaluation, but took the mutations from the `.txt` file.

For sample size 15 and 20, the running times were too large, for example their iterative method did not improve the solution for one dataset (out of 100) during several days. In general, the running time was not stable, ranging from several hours to several days per dataset.

CITUP Since our simulated trees have 10 clones, we set the maximum number of clusters to 10 (parameter `-c`), up from the default value 5. CITUP has two operating, modes Quadratic Integer Programming (QIP) and a heuristic iterative method. In our experiments, the QIP method did indeed give consistently better results, so we reported only this one.

CITUP requires the VAF values to be lower than 0.5 (although in our experiments it ran also for VAF values up to 0.6), otherwise it cannot run. For read coverage 100, many mutations have such high VAF values, and as such we cannot report CITUP results for read coverage 100. (The error given in console was *KeyError: 'Objective_value'*.)

Treeomics We used all default parameters. For sample sizes 15 and 20, Treeomics did not run (it was killed by the operating system with only the message `killed`).

PASTRI We tried running also PASTRI [8], but as we will describe below, we could not run it. We tried to contact the authors by e-mail and opened an issue in the github repository[1], but we did not get any reply. At the time of writing, there are two other persons indicating the same issue on github.

Recall that PASTRI needs an input proposal for the parameters α, β. In the PASTRI paper, they are obtained with SciClone [5]. First, we tried to run SciClone on the example in the PASTRI

[1] `https://github.com/raphael-group/PASTRI/issues/3`

repository[2], but did not get from SciClone the same proposal as indicated by PASTRI.[3] Second, we ran SciClone on our simulated data, and then ran PASTRI on the proposal obtained form SciClone. However, PASTRI did not run, and reported the error: `ValueError: zero-size array to reduction operation maximum which has no identity`.

1.2 Additional results on simulated data

Table 1. Results of MIPUP, LICHeE, Treeomics (fraction of AD pairs present in the output trees), for 5 samples taken from trees with 20 nodes, 200 mutations, and 30 nodes, 300 mutations. We also varied the number d of lost mutations from 0 (perfect phylogeny assumption) to 9.

		MIPUP			LICHeE	Treeomics		
tree nodes / mutations	d	Best	Avg	Std		Best	Avg	Std
20 / 200	0	0.595	**0.574**	0.027	0.522	0.586	0.564	0.020
	1	0.561	**0.538**	0.028	0.491	0.549	0.529	0.018
	2	0.500	**0.477**	0.029	0.414	0.492	0.474	0.016
	3	0.494	0.466	0.034	0.418	0.494	**0.477**	0.016
	4	0.486	0.486	0.030	0.427	0.522	**0.497**	0.022
	5	0.427	0.403	0.026	0.329	0.447	**0.430**	0.015
	6	0.399	0.373	0.028	0.324	0.415	**0.393**	0.019
	7	0.351	0.326	0.027	0.265	0.396	**0.374**	0.019
	8	0.322	0.304	0.024	0.256	0.350	**0.334**	0.014
	9	0.304	0.287	0.018	0.222	0.332	**0.316**	0.014
30 / 300	0	0.476	**0.453**	0.023	0.407	0.465	0.451	0.013
	1	0.501	0.477	0.027	0.435	0.497	**0.482**	0.014
	2	0.460	0.436	0.026	0.398	0.458	**0.443**	0.013
	3	0.438	0.420	0.021	0.360	0.443	**0.427**	0.015
	4	0.444	0.423	0.026	0.372	0.448	**0.433**	0.014
	5	0.380	0.360	0.021	0.308	0.388	**0.374**	0.012
	6	0.401	0.382	0.020	0.307	0.415	**0.398**	0.015
	7	0.362	0.341	0.023	0.291	0.376	**0.364**	0.012
	8	0.333	0.312	0.021	0.255	0.356	**0.338**	0.015
	9	0.343	0.321	0.022	0.262	0.358	**0.345**	0.012

[2] `https://github.com/raphael-group/PASTRI/tree/master/example`
[3] `https://github.com/raphael-group/PASTRI/blob/master/example/Example.proposal`

Table 2. Results for $d \in \{3, 4, 5\}$ (not included in Table 2 in the main paper).

d=3		MIPUP			LICHeE	Treeomics			CITUP			Ances
m	coverage	Best	Avg	Std		Best	Avg	Std	Best	Avg	Std	Tree
5	100	0.544	0.521	0.04	0.440	0.553	**0.533**	0.02				0.074
	1000	0.521	0.498	0.05	0.428	0.557	**0.522**	0.03		0.343	0.13	0.052
	10000	0.496	0.479	0.04	0.419	0.522	**0.486**	0.03		0.325	0.10	0.041
10	100	0.606	0.591	0.03	0.482	0.670	**0.641**	0.03				0.015
	1000	0.622	0.597	0.05	0.521	0.671	**0.630**	0.04		0.318	0.14	0.013
	10000	0.584	0.569	0.03	0.506	0.661	**0.590**	0.07		0.323	0.02	0.011
15	100	0.581	**0.566**	0.03	0.470							
	1000	0.593	**0.581**	0.05	0.546							
	10000	0.625	**0.620**	0.03	0.560							
20	100	0.575	**0.564**	0.03	0.467							
	1000	0.593	**0.589**	0.04	0.549							
	10000	0.595	**0.589**	0.04	0.552							

d=4		MIPUP			LICHeE	Treeomics			CITUP			Ances
m	coverage	Best	Avg	Std		Best	Avg	Std	Best	Avg	Std	Tree
5	100	0.471	0.445	0.05	0.369	0.511	**0.488**	0.02				0.039
	1000	0.453	0.427	0.04	0.351	0.515	**0.486**	0.03	0.317	0.291	0.13	0.042
	10000	0.509	0.479	0.06	0.383	0.516	**0.485**	0.03	0.380	0.351	0.12	0.042
10	100	0.544	0.520	0.04	0.405	0.643	**0.615**	0.03				0.015
	1000	0.558	0.538	0.04	0.468	0.617	**0.579**	0.04	0.339	0.338	0.02	0.012
	10000	0.526	0.504	0.04	0.454	0.631	**0.549**	0.09	0.309	0.304	0.06	0.008
15	100	0.513	**0.503**	0.03	0.415							
	1000	0.536	**0.524**	0.04	0.488							
	10000	0.503	**0.493**	0.03	0.452							
20	100	0.470	**0.463**	0.02	0.380							
	1000	0.520	**0.515**	0.02	0.490							
	10000	0.518	**0.512**	0.03	0.476							

d=5		MIPUP			LICHeE	Treeomics			CITUP			Ances
m	coverage	Best	Avg	Std		Best	Avg	Std	Best	Avg	Std	Tree
5	100	0.383	0.361	0.04	0.285	0.450	**0.424**	0.02				0.036
	1000	0.406	0.380	0.04	0.310	0.483	**0.443**	0.03	0.279	0.257	0.11	0.032
	10000	0.423	0.394	0.05	0.314	0.504	**0.467**	0.03	0.235	0.222	0.05	0.051
10	100	0.436	0.419	0.03	0.352	0.592	**0.549**	0.04				0.010
	1000	0.479	0.451	0.05	0.391	0.581	**0.533**	0.04	0.250	0.243	0.06	0.010
	10000	0.424	0.409	0.03	0.351	0.538	**0.474**	0.05	0.225	0.225	0.00	0.008
15	100	0.428	**0.414**	0.03	0.354							
	1000	0.394	**0.384**	0.03	0.358							
	10000	0.407	**0.396**	0.03	0.355							
20	100	0.381	**0.376**	0.02	0.324							
	1000	0.425	**0.422**	0.03	0.389							
	10000	0.394	**0.391**	0.02	0.366							

Table 3. Results for $d \in \{6, 7, 8\}$ (not included in Table 2 in the main paper).

d=6		MIPUP			LICHeE	Treeomics			CITUP			Ances
m	coverage	Best	Avg	Std		Best	Avg	Std	Best	Avg	Std	Tree
5	100	0.351	0.330	0.03	0.263	0.431	**0.403**	0.02				0.040
	1000	0.365	0.340	0.04	0.266	0.439	**0.412**	0.02	0.205	0.187	0.06	0.030
	10000	0.361	0.330	0.04	0.277	0.458	**0.433**	0.02	0.197	0.181	0.07	0.015
10	100	0.359	0.340	0.03	0.277	0.509	**0.475**	0.03				0.009
	1000	0.385	0.352	0.06	0.308	0.557	**0.517**	0.04	0.190	0.187	0.02	0.006
	10000	0.382	0.365	0.03	0.326	0.587	**0.518**	0.07	0.180	0.179	0.00	0.008
15	100	0.365	**0.348**	0.04	0.300							
	1000	0.314	**0.304**	0.04	0.274							
	10000	0.348	**0.339**	0.03	0.316							
20	100	0.345	**0.338**	0.02	0.280							
	1000	0.377	**0.373**	0.03	0.339							
	10000	0.364	**0.361**	0.04	0.340							

d=7		MIPUP			LICHeE	Treeomics			CITUP			Ances
m	coverage	Best	Avg	Std		Best	Avg	Std	Best	Avg	Std	Tree
5	100	0.287	0.260	0.04	0.208	0.383	**0.348**	0.03				0.016
	1000	0.272	0.247	0.04	0.188	0.393	**0.359**	0.03	0.183	0.162	0.06	0.017
	10000	0.298	0.278	0.03	0.218	0.402	**0.370**	0.03	0.201	0.187	0.05	0.012
10	100	0.321	0.297	0.04	0.252	0.468	**0.435**	0.03				0.007
	1000	0.344	0.327	0.03	0.274	0.521	**0.472**	0.05	0.158	0.158	0.00	0.009
	10000	0.294	0.277	0.03	0.250	0.495	**0.425**	0.06	0.126	0.126	0.00	0.004
15	100	0.283	**0.272**	0.02	0.248							
	1000	0.313	**0.303**	0.03	0.281							
	10000	0.307	**0.300**	0.03	0.274							
20	100	0.291	**0.288**	0.01	0.235							
	1000	0.278	**0.274**	0.03	0.249							
	10000	0.284	**0.281**	0.02	0.260							

d=8		MIPUP			LICHeE	Treeomics			CITUP			Ances
m	coverage	Best	Avg	Std		Best	Avg	Std	Best	Avg	Std	Tree
5	100	0.281	0.260	0.03	0.198	0.361	**0.336**	0.02				0.028
	1000	0.280	0.251	0.04	0.209	0.358	**0.322**	0.03	0.100	0.132	0.03	0.018
	10000	0.237	0.216	0.04	0.173	0.345	**0.314**	0.03	0.105	0.146	0.06	0.012
10	100	0.249	0.233	0.02	0.205	0.411	**0.385**	0.03				0.007
	1000	0.249	0.229	0.03	0.215	0.435	**0.389**	0.04	0.100	0.142	0.00	0.005
	10000	0.245	0.227	0.02	0.201	0.494	**0.430**	0.05	0.108	0.164	0.01	0.004
15	100	0.241	**0.234**	0.02	0.200							
	1000	0.247	**0.234**	0.04	0.216							
	10000	0.227	**0.218**	0.03	0.203							
20	100	0.232	**0.231**	0.00	0.200							
	1000	0.235	**0.231**	0.03	0.210							
	10000	0.253	**0.251**	0.01	0.236							

2 Further details on real data

2.1 Running the tools

On the first three real datasets [3, 1, 2], we ran LICHeE as instructed at [6] and as done in [7]. In particular, we used the same two thresholds t_0 and t_1 for declaring an SSNV to be 0 (if its VAF value is smaller than t_0), 1 (if its VAF value is greater or equal to t_1), or in between these thresholds (in the 'greyzone'). The threshold values chosen by LICHeE follow those used in the original publications. MIPUP uses only one threshold t and we always chose $t = t_1$. On the leiomyomas datasets, for MIPUP we used the threshold $t = 0.05$, as done in [4]. LICHeE did not find any tree for $t_0 = t_1 = 0.05$, thus we set $t_0 = 0.05$ and $t_1 = 0.06$.

For MIPUP, on the first three datasets [3, 1, 2] we did not filter weak SSNVs (i.e., we have $k = 1$, recall the Implementation section), given the relatively small number of SSNVs (i.e., columns), see Table 4. On the leiomyomas dataset, having an order of magnitude more SSNVs, we filtered weak SSNVs with $k = 2$. Table 4 also shows how big are the matrices corresponding to the real samples. It also shows by how much the number of rows increases in the conflict-free output of the MCRS problem.

Table 4. An overview of the datasets and of the output of MIPUP; "dist." stands for "distinct". Bold rows correspond to input matrices that are *not* already conflict-free.

Dataset	Input	Input matrix #rows	#cols	#dist. cols	MIPUP #rows
ccRCC [3]	EV003	8	42	5	8
	EV005	7	73	7	7
	EV006	9	66	5	9
	**EV007**	**8**	**53**	**8**	**10**
	RHM002	5	48	8	5
	RHM004	6	124	10	6
	**RHM008**	**8**	**77**	**10**	**10**
	**RK26**	**11**	**61**	**10**	**12**
HGSC [1]	case1	4	69	4	4
	case2	4	49	5	4
	case3	3	29	2	3
	**case4**	**10**	**57**	**4**	**14**
	**case5**	**6**	**124**	**10**	**10**
	case6	2	35	1	2
xenoengraftment [2]	**SA501-X1X2X4**	**3**	**180**	**5**	**4**
leiomyomas [4]	**MY21**	**4**	**1475**	**9**	**5**

2.2 Additional results on real data

Below, we discuss the results of MIPUP and LICHeE on three other real samples.

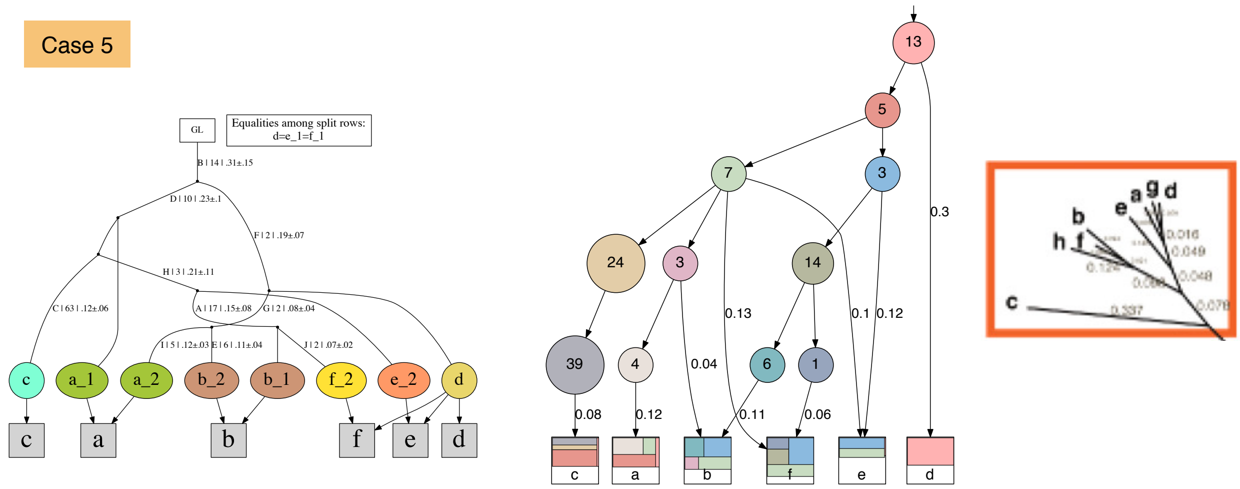

Fig. 1. Results of MIPUP (left), LICHeE (middle), and the original tree (right) on input Case 5 from [1].

In dataset Case 5 from [1] (Fig. 1), LICHeE reports each sample **b,f,e** to be a mixture of two phylogeny nodes, whereas MIPUP reports that so is sample **a**. Even though the original phylogeny from [1] indicates no mixture in the samples, a more detailed analysis performed by [7] suggests that **b,f,e** are indeed mixed samples. In fact, as [7] notice, the trees from [1] were obtained using a neighbor-joining algorithm that "produces trees with lack of evidential support in some branches." The trees output by LICHeE and MIPUP have a supporting SSNV group on each node or edge.

Moreover, we investigated why MIPUP reports that sample **a** is a mixture of two phylogeny leaves, as opposed to LICHeE. One reason is that sample **a** contains two mutations (on the tree edge labeled F) that are present in samples **b** and **d** and in no others, combination which is not possible in LICHeE's tree. We then discovered that these two SSNVs in edge F were considered

weak by LICHeE and filtered out, because it used a more permissive threshold $k = 3$, recall the Implementation section. We then ran LICHeE with the default value $k = 2$, but it did not find any valid trees (partially explaining the choice $k = 3$). We also noticed that MIPUP reports a mutation in gene TRIM40 only in sample **a** (on the tree edge labeled I). This was also concluded in [1, Fig. S6]. However, LICHeE reports that it is present also in sample **b**. Its VAF value is 0.177 in sample **b**, which fits the grayzone [0.1..0.4) of LICHeE for this dataset. Thus, due to some internal choices, LICHeE converts this value into 1.

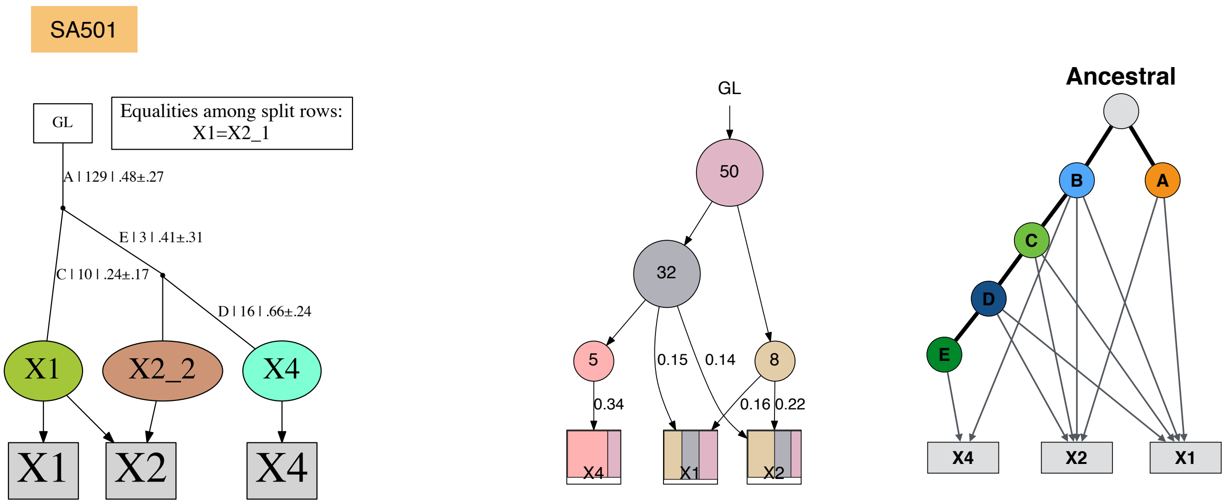

Fig. 2. Results of MIPUP (left), LICHeE (middle), and the original tree (right) on input SA501 from [2].

In dataset SA501 from [2] (Fig. 2), both MIPUP and LICHeE support the original predictions of [2] that sample X4 contains mutations private to it. LICHeE predicts that X1 and X2 are combinations of the same nodes of the phylogeny. Samples X1 and X2 coincide also in the trees of MIPUP, except for three mutations private to X2 (the edge labeled E). They were filtered out by LICHeE because one was considered weak, and the other two had VAF values 0.79, and 0.84, respectively, which was considered invalid, as being greater than a default threshold 0.6. However, these mutations were validated by targeted deep amplicon reseqencing [2, Table S5].

We then observed that LICHeE ran with a very permissive threshold for filtering out weak SSNVs, $k = 6$. We also ran MIPUP with $k = 6$ and observed that these three private mutations were also filtered out, and nodes X1 and X2_2 were merged into one (making X1 and X2 have the same mutations). We also ran LICHeE with $k = 1$, and now obtained an opposite picture (see Fig. 3): X1 and X2 had the same mutations, except for one mutation private to X1. This is another example of how the parameters controlling the various tools can affect the predictions.

In dataset MY21 from [4] (Fig. 4), both MIPUP and LICHeE give predictions compatible with the biological interpretations presented in [4]: sample m2 initially diverges, then a split in the phylogeny happens and sample m4 is a combination of these two subpopulations. Each of these subpopulations further acquires mutations, and then are sequenced in samples m1 and m3, respectively.

We show below the trees output by MIPUP and LICHeE on other real samples.

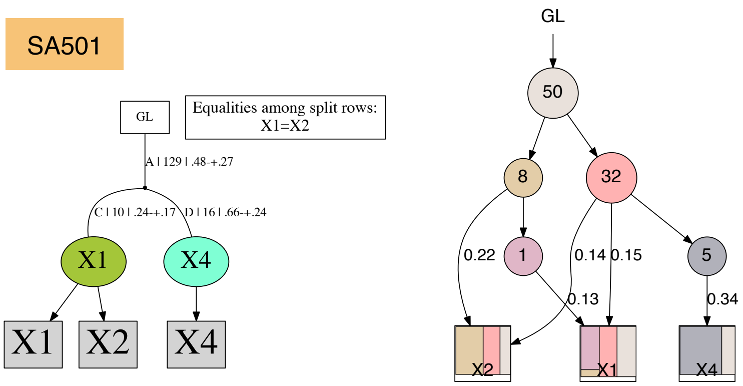

Fig. 3. Input SA501 from [2]. Left: the result of MIPUP after filtering the weak SSNVs with appearance threshold $k = 6$. Samples X1 and X2 have the same mutations. Right: the result of LICHeE with threshold $k = 1$ (option minClusterSize=1). Sample X1 has one private mutation.

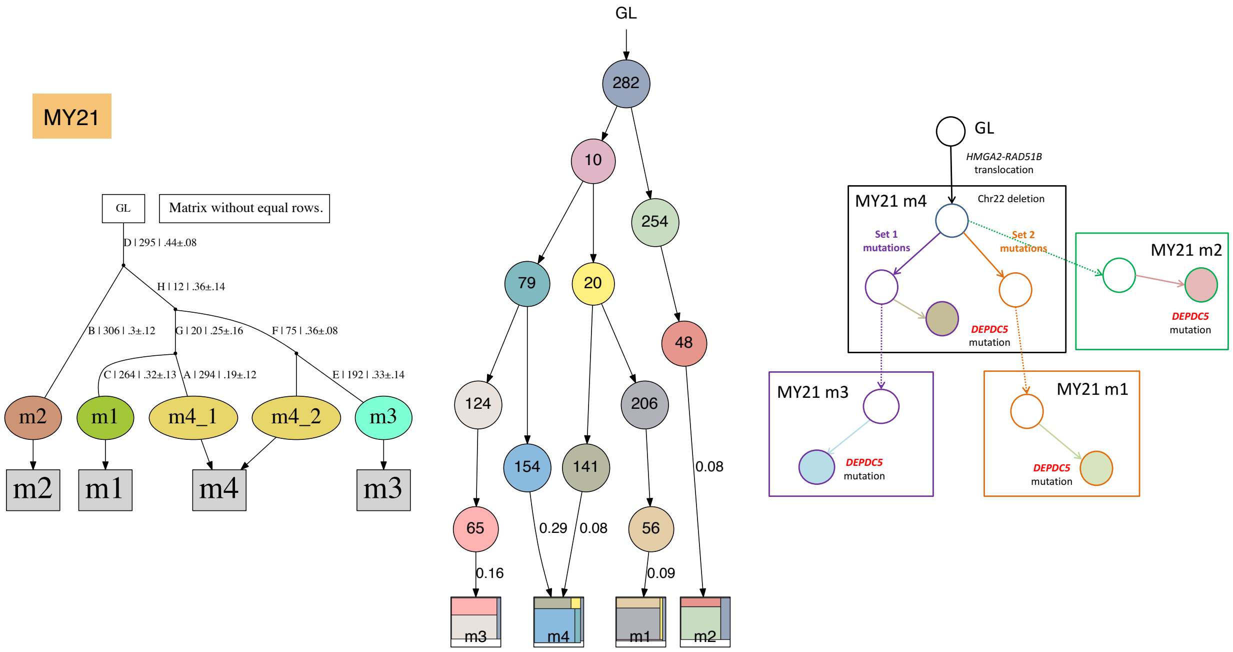

Fig. 4. Results of MIPUP (left), LICHeE (middle), and the original tree (right) on input MY21 from [4].

References

1. Ali Bashashati, Gavin Ha, Alicia Tone, Jiarui Ding, Leah M Prentice, Andrew Roth, Jamie Rosner, Karey Shumansky, Steve Kalloger, Janine Senz, Winnie Yang, Melissa McConechy, Nataliya Melnyk, Michael Anglesio, Margaret TY Luk, Kane Tse, Thomas Zeng, Richard Moore, Yongjun Zhao, Marco A Marra, Blake Gilks, Stephen Yip, David G Huntsman, Jessica N McAlpine, and Sohrab P Shah. Distinct evolutionary trajectories of primary high-grade serous ovarian cancers revealed through spatial mutational profiling. *The Journal of Pathology*, 231(1):21–34, 2013.
2. Peter Eirew, Adi Steif, Jaswinder Khattra, Gavin Ha, Damian Yap, Hossein Farahani, Karen Gelmon, Stephen Chia, Colin Mar, Adrian Wan, Emma Laks, Justina Biele, Karey Shumansky, Jamie Rosner, Andrew McPherson,

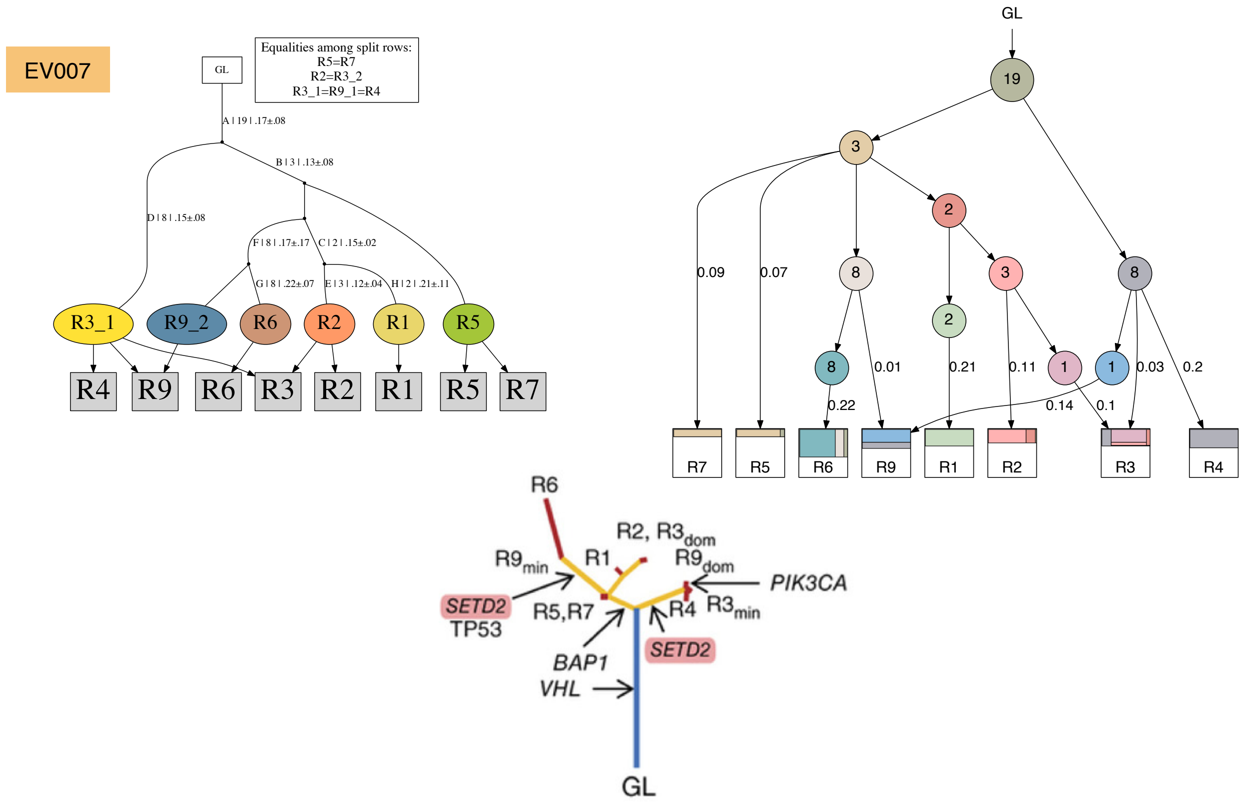

Fig. 5. Results of MIPUP (top left), LICHeE (top right), and the original tree from [3] (bottom) on input EV007.

Cydney Nielsen, Andrew J. L. Roth, Calvin Lefebvre, Ali Bashashati, Camila de Souza, Celia Siu, Radhouane Aniba, Jazmine Brimhall, Arusha Oloumi, Tomo Osako, Alejandra Bruna, Jose L. Sandoval, Teresa Algara, Wendy Greenwood, Kaston Leung, Hongwei Cheng, Hui Xue, Yuzhuo Wang, Dong Lin, Andrew J. Mungall, Richard Moore, Yongjun Zhao, Julie Lorette, Long Nguyen, David Huntsman, Connie J. Eaves, Carl Hansen, Marco A. Marra, Carlos Caldas, Sohrab P. Shah, and Samuel Aparicio. Dynamics of genomic clones in breast cancer patient xenografts at single-cell resolution. *Nature*, 518(7539):422–426, 02 2015.
3. Marco Gerlinger, Stuart Horswell, James Larkin, Andrew J Rowan, Max P Salm, Ignacio Varela, Rosalie Fisher, Nicholas McGranahan, Nicholas Matthews, Claudio R Santos, Pierre Martinez, Benjamin Phillimore, Sharmin Begum, Adam Rabinowitz, Bradley Spencer-Dene, Sakshi Gulati, Paul A Bates, Gordon Stamp, Lisa Pickering, Martin Gore, David L Nicol, Steven Hazell, P Andrew Futreal, Aengus Stewart, and Charles Swanton. Genomic architecture and evolution of clear cell renal cell carcinomas defined by multiregion sequencing. *Nat Genet*, 46(3):225–233, 03 2014.
4. Miika Mehine, Hanna-Riikka Heinonen, Nanna Sarvilinna, Esa Pitkänen, Netta Mäkinen, Riku Katainen, Sari Tuupanen, Ralf Bützow, Jari Sjöberg, and Lauri A. Aaltonen. Clonally related uterine leiomyomas are common and display branched tumor evolution. *Human Molecular Genetics*, 24(15):4407, 2015.
5. Christopher A. Miller et al. SciClone: Inferring Clonal Architecture and Tracking the Spatial and Temporal Patterns of Tumor Evolution. *PLoS Comput Biol*, 10(8):e1003665+, August 2014.
6. Victoria Popic. https://github.com/viq854/lichee/tree/master/lichee/data, 2016.
7. Victoria Popic, Raheleh Salari, Iman Hajirasouliha, Dorna Kashef-Haghighi, Robert B. West, and Serafim Batzoglou. Fast and scalable inference of multi-sample cancer lineages. *Genome Biology*, 16(1):1–17, 2015.
8. Gryte Satas and Benjamin J. Raphael. Tumor phylogeny inference using tree-constrained importance sampling. *Bioinformatics*, 33(14):i152–i160, 2017.

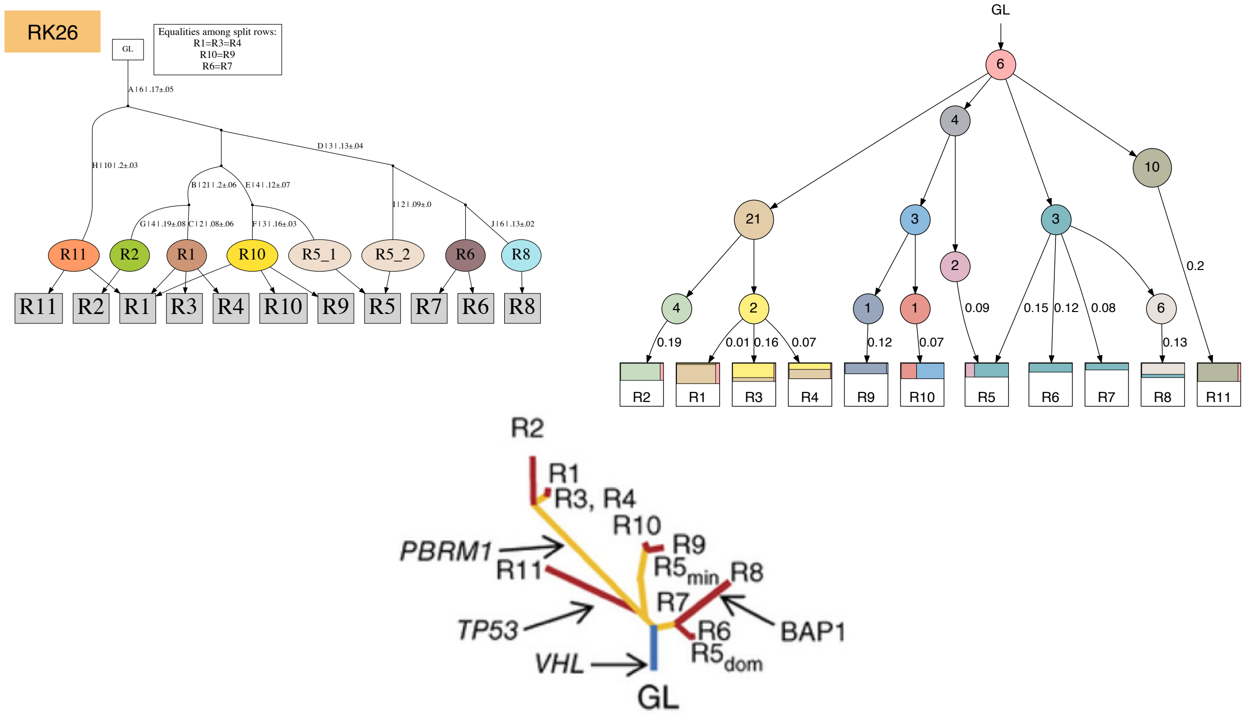

Fig. 6. Results of MIPUP (top left), LICHeE (top right), and the original tree from [3] (bottom) on input RK26.

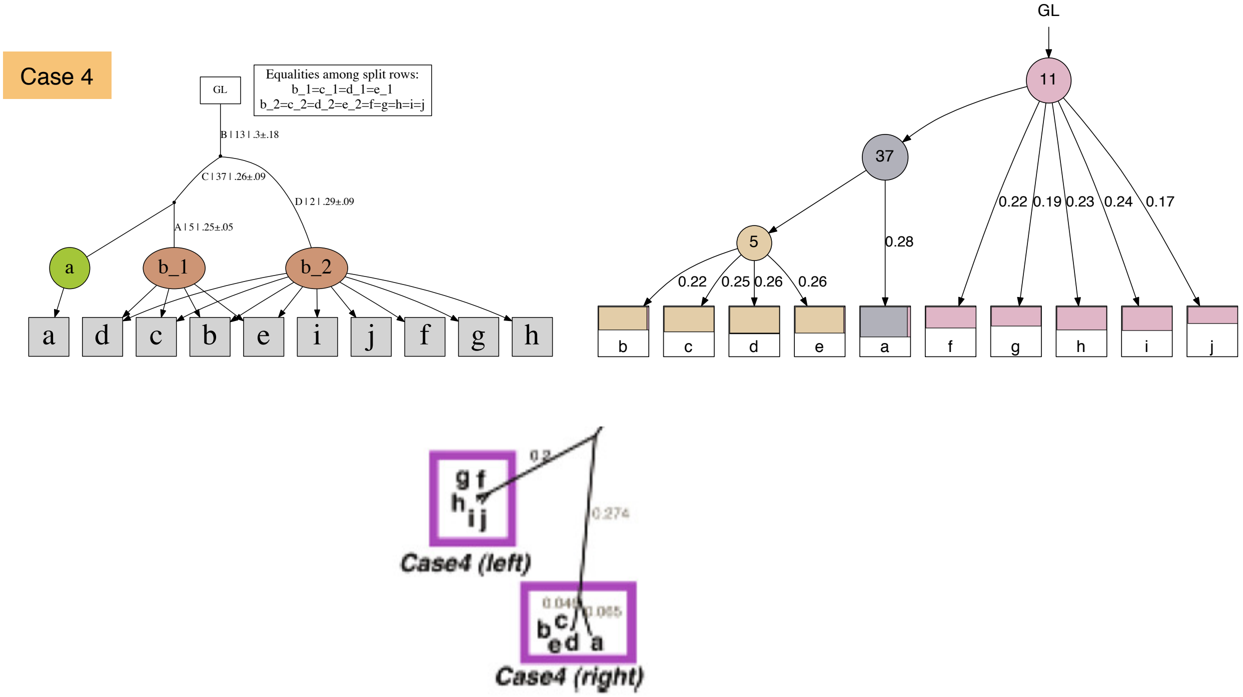

Fig. 7. Results of MIPUP (top left), LICHeE (top right), and the original tree from [1] (bottom) on input Case 4.
